# Musical Agency during Physical Exercise Decreases Pain

**DOI:** 10.3389/fpsyg.2017.02312

**Published:** 2018-01-17

**Authors:** Thomas H. Fritz, Daniel L. Bowling, Oliver Contier, Joshua Grant, Lydia Schneider, Annette Lederer, Felicia Höer, Eric Busch, Arno Villringer

**Affiliations:** ^1^Department of Neurology, Max Planck Institute for Human Cognitive and Brain Sciences, Leipzig, Germany; ^2^Department of Nuclear Medicine, University of Leipzig, Leipzig, Germany; ^3^Institute for Psychoacoustics and Electronic Music, University of Ghent, Ghent, Belgium; ^4^Department of Cognitive Biology, University of Vienna, Vienna, Austria

**Keywords:** pain, musical agency, cold pressor test, endurance, sport, endorphin

## Abstract

**Objectives:** When physical exercise is systematically coupled to music production, exercisers experience improvements in mood, reductions in perceived effort, and enhanced muscular efficiency. The physiology underlying these positive effects remains unknown. Here we approached the investigation of how such musical agency may stimulate the release of endogenous opioids indirectly with a pain threshold paradigm.

**Design:** In a cross-over design we tested the opioid-hypothesis with an indirect measure, comparing the pain tolerance of 22 participants following exercise with or without musical agency.

**Method:** Physical exercise was coupled to music by integrating weight-training machines with sensors that control music-synthesis in real time. Pain tolerance was measured as withdrawal time in a cold pressor test.

**Results:** On average, participants tolerated cold pain for ~5 s longer following exercise sessions with musical agency. Musical agency explained 25% of the variance in cold pressor test withdrawal times after factoring out individual differences in general pain sensitivity.

**Conclusions:** This result demonstrates a substantial pain reducing effect of musical agency in combination with physical exercise, probably due to stimulation of endogenous opioid mechanisms. This has implications for exercise endurance, both in sports and a multitude of rehabilitative therapies in which physical exercise is effective but painful.

## Introduction

### Musical agency

Control over musical sound (musical agency) is experienced by singers and musicians on a regular basis during music performance. Such experience of agency in music seems to be perceived as highly relevant to the performers, who are highly capable of recognizing their own performances at later time points (Repp and Knoblich, 2004; Keller et al., 2007; Repp and Keller, 2010; Sevdalis and Keller, 2014). Non-musicians, however, can also experience musical agency, especially when the “musical instruments” are appropriately adapted to their capabilities.

### Jymmin-paradigm

In a paradigm using weight training machines with musical feedback, muscular effort can be closely coupled to musical sound, giving any participant the opportunity to experience a possibility for musical expression and the broad range of positive psychological and physiological effects that follow (Fritz et al., 2013a,b, 2015). This approach, dubbed *Jymmin* (gym + jammin), combines exercise machines designed for weight training with sensors to control music production software in real time. Integrating exercise and musical agency in this way stimulates improvements in mood, reductions in perceived effort, and enhanced muscular efficiency compared to control conditions where participants engaged in the same exercises but without their movements affecting the music (Fritz et al., 2013a,b). On a physiological level, the positive effects of this intervention on mood (as measured on mood subscale of the Multidimensional Mood Questionnaire Steyer et al., 1997) may result from a release of endogenous opioids in the central nervous system.

### Opioids and pain sensitivity

Physical exercise is known to be an effective trigger of opioid activity (Sforzo, 1989; Droste et al., 1991; Goldfarb and Jamurtas, 1997; Boecker et al., 2008), which has also been implicated in mediating improved mood and decreased pain sensitivity when listening to/participating in music (Västfjäll, 2001; Dunbar et al., 2012; Roy et al., 2012). With regard to hypoalgesic effects of physical exercise, previous meta-analytic data from healthy participants suggested that isometric exercise over durations ≥5 min can have large effects on pain sensitivity (*d* = 1.74; *SD* = 0.75; Naugle et al., 2012). Because there is also a relation of endorphin levels and grooming in non-human primates, it has been suggested that music-making may represent a form of social interaction that allows for expansion of cohesive groups beyond the limits imposed by the requirements of one-to-one grooming interactions thus for example facilitating bonding even in large choirs (Weinstein et al., 2016).

The previously observed effects of Jymmin on mood give rise to the hypothesis that temporal coupling between muscular effort and musical sound is a particularly effective trigger for the release of endogenous opioids, highlighting the potential utility of musical agency in sports endurance and rehabilitative therapy.

Assessment of endogenous opioids in the central nervous system is complicated by the fact that they do not easily cross the blood brain barrier (Dearman and Francis, 1983; Kalin and Loevinger, 1983). Existing methods for direct measurement are highly invasive, requiring intravenous injection of radioactive tracers or the extraction of cerebrospinal fluid (Hosobuchi et al., 1979; Boecker et al., 2008). Here we use pain sensitivity as a proxy for central opioid levels. This approach is common in human behavioral research (Zillmann et al., 1993; Depue and Morrone-Strupinsky, 2005; Cohen et al., 2010; Dunbar et al., 2012) and supported by the link between analgesia and opioids in medicine (Mayer and Hayes, 1975; Hosobuchi et al., 1979; Bandura et al., 1987; Fields, 2000; Zubieta et al., 2001, 2003).

Inter-individual variability in experimentally determined pain sensitivity has been shown to be substantial (Dionne et al., 2005; Fillingim, 2005). Medical studies of chronic opioid abuse have shown that down-regulation of the endogenous opioid system (mu receptors) can result in increased pain sensitivity (hyperalgesia), as well as painful responses to normally non-painful stimuli (allodynia)(Sprouse-Blum et al., 2010). These results imply that individual pain sensitivity relates to the activity and regulation of endogenous opioids, modulating the effects of endorphin release, such that increased pain sensitivity is associated with decreases in hypoalgesic effects of endorphin. Because inter-individual variability in baseline pain sensitivity can be substantial, it is critical to consider this important source of variation in pain research (Dionne et al., 2005; Fillingim, 2005).

An approximation of individual pain sensitivity can be achieved with the Pain Sensitivity Questionnaire (PSQ). It is a self-rating instrument that involves making pain intensity ratings of daily life situations (Ruscheweyh et al., 2009). It includes two sub-scores, one based on items referring to mildly painful situations (the PSW-minor), and one based on items referring to moderately painful situations (the PSQ-moderate). Correlations between PSQ scores and ratings of experimentally induced pain are highest for the PSQ-minor score, indicating that it is a better predictor of individual pain sensitivity than either the PSQ-moderate or PSQ-total (perhaps because mildly painful situations are perceived to be manageable). The PSQ may thus be reduced to the PSQ-minor items without losing relevant information (Ruscheweyh et al., 2009, 2012).

### Relevance of study, research questions

In the context of the present study, it is important to note that regardless of the precise mechanism underlying changes in pain sensitivity, it is clear that a method to systematically reduce pain associated with physical exercise would be of high medical relevance, with applications ranging from sports medicine to the prevention of injury and rehabilitation in non-athletes. Indeed, patients suffering from various forms of physical injury and/or neural disorders, as well as many elderly often display activity avoidance, rejecting highly effective physical exercise therapy because for them it is painful (Geisser et al., 2000; Mannerkorpi and Iversen, 2003; Crombie et al., 2004).

Here we investigated an influence of musical agency during physical exercise on pain threshold. We hypothesized that the pain threshold is heightened after performing physical exercise with musical agency as compared to the same type of physical exercise performance with passive music listening. We tested this hypothesis in a cross-over design in which every participant performed both, the experimental and control condition.

## Materials and methods

### Participants

Twenty-two healthy human participants took part in this study (12 female; mean age = 25 years; age range = 21–29; *SD* = 2.41). None of the participants had mental or motor disorders, and none had a history of cardiovascular disease or chronic pain based on self-report. Exclusion criteria also included professional musicians, athletes or body builders. Participants were run in pairs. The association of participants into pairs was random, and an examination of influences of gender was not addressed in the current study. Participants received monetary compensation after completion of the second-day of the experiment. Ethical approval was obtained from the Ethics Committee of the University of Leipzig, and the experiment was carried out in accordance with the guidelines of the local institutional review board at the University of Leipzig. Informed consent was obtained from all participants after explanation of the experiment and the risks involved in participation. Participants were also made aware that they could withdraw from the study at any time without further consequences.

Three of the 22 participants were excluded from the analysis because they either did not show up for the second session, the cooling-system for the cold pressor task failed, or they completed the cold pressor test without reporting pain. One participant (whose partner did not turn up on the second day) had to perform with a substitute partner [with the same gender (female) as the original partner and within the age range of the participant group] allowing us to retain her data in the analysis. Accordingly, the statistical analysis included 19 participants (10 male; mean age = 25.11 years; age range = 21–29; *SD* = 2.45).

### Experimental design

Participants worked out in groups of two (randomly assigned to group, no prior relationship). Each pair participated in two experimental conditions in a within-subjects design. Both conditions involved operating exercise machines while listening to music, followed by administration of a CPT. In the *agency* condition exercise movements controlled the music. In the *no-agency* condition exercise movements did not control the music, which was instead a recording of the *agency* condition of a different participant pair (with the exception of the first pair, who listened to a recording of their own *agency* condition in the *no-agency* condition). Each condition was run in a separate session on a separate day with at least one night and maximum of nine nights between sessions. The order in which participants performed the conditions was counterbalanced across pairs (allocation ratio 1:1). In addition to controlling for traditional order effects, this counterbalancing was also aimed at controlling for potential biases in pain sensitivity based on having previous experience with the CPT. All experimental data were acquired within 2 weeks.

### Musical feedback equipment

**Figure 1:**
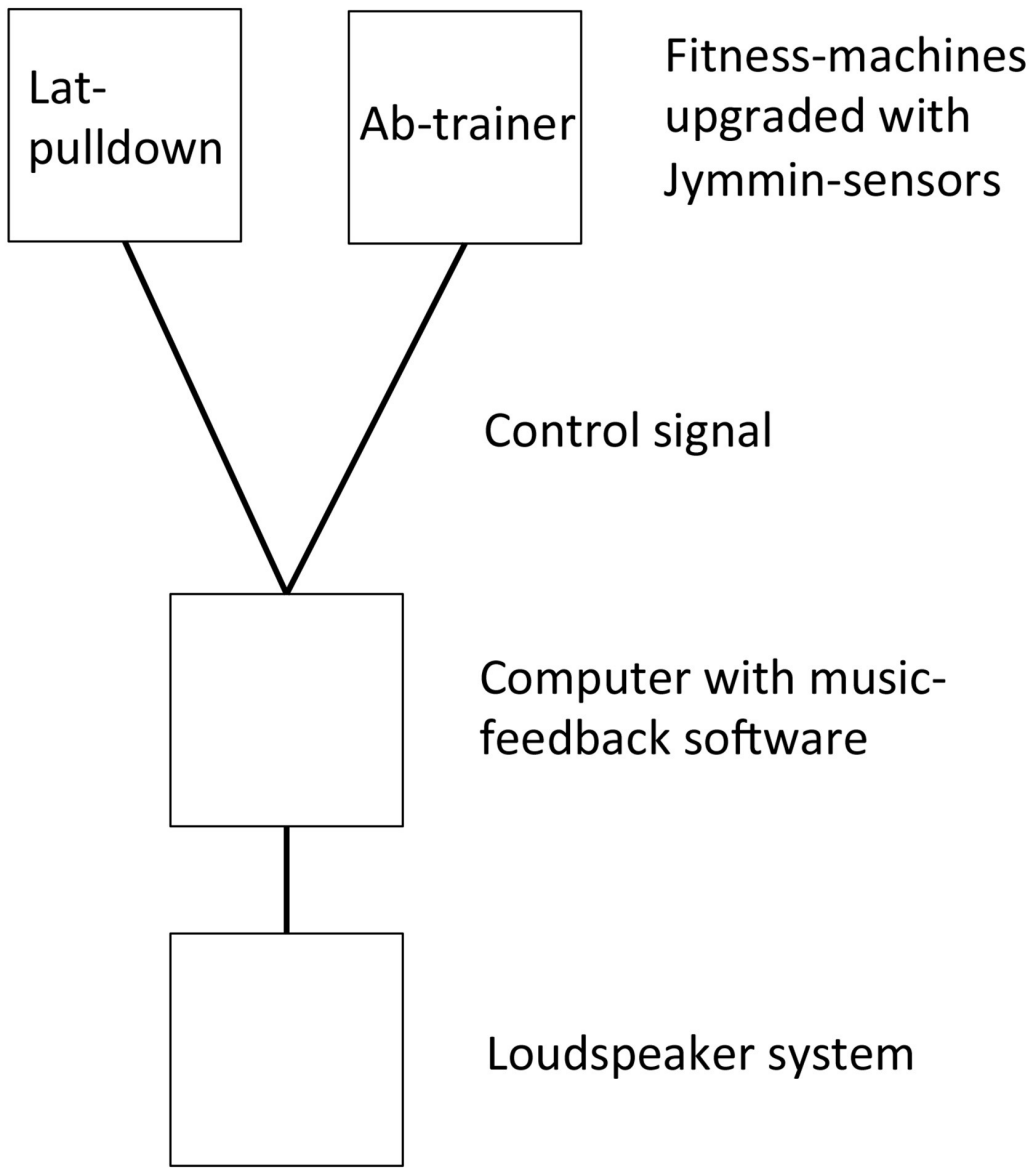
Schematic illustration of experimental setup.

Two physical exercise machines were used in this study, a cable lat pulldown machine and an abdominal trainer, both of which have been used in previous music feedback studies with a similar paradigm (Fritz et al., 2013a,b, 2015). These standard fitness machines allow for guided movements designed to train specific muscle groups. The cable lat pulldown machine consisted of a metal bar attached via cable and pulley to an adjustable weight stack that was moved vertically. Using an underhand (supinated) grip, participants pulled the metal bar down to lift the stack, bilaterally training the latissimi dorsi and biceps muscles. Hypoalgesic effects of biceps training has been described previously (Naugle et al., 2012). The abdominal trainer used the participant's own body mass as resistance to a “sit-up” motion designed to train the abdominal muscles. Because previous work suggested that participants were quickly exhausted by this exercise, they were allowed to use their arms for assistance via a semi-circular handle attached to the machine's backrest. Nevertheless, it was not possible to execute the sit-up motion without abdominal engagement. Sensors placed on the weight stack on the lat-pulldown and on the back support of the abdominal trainer transduced their configuration into digital signals used to manipulate music under the control of computer software (Ableton Live 8™; see Figure 1). Movement related weight shift parameters during both the experimental condition (musical feedback) and control condition were monitored and recorded.

### Music

The music consisted of a set of synchronized repeating musical loops at a tempo of 130 beats per minute, which in previous experiments was perceived as appropriate for physical exercise by participants (Fritz et al., 2013a,b, 2015). The music consisted of a simple 4:4 drum beat (bass drum, hi-hat, and cymbals), a bass line (electric bass guitar), and a continuously fluctuating melody line (synthesizer) that could be varied in pitch. In the *agency* condition (described below), the movements of each machine were mapped to modulate different acoustic parameters. The pull-down machine controlled the cutoff frequencies of bandpass filters on the drum beat and bass line loops such that the more the bar was pulled down, the more the frequencies in the higher part of the spectrum were audible (an effect typically used by disc jockeys to modify sound spectra). The abdominal trainer controlled the pitch of the melody line such that more abdominal engagement resulted in synthesizer sounds with higher pitch. These effects were calibrated so that audible changes in the music could be created along the entire movement range. Audio was presented over loudspeakers in both conditions (amplitude = ~65 dBA). The non-agency condition included music recordings from the agency conditions of other participants (except the first group who listened to a music piece performed during their own agency condition), which could not be modified by participants. This was done to avoid that effects could be due to different basic acoustical features of the music during the agency and non-agency conditions.

Recently it was shown that physical exertion by participants in music making results in a positive perceptual bias that increases the aesthetic quality of music (the “band effect”; Fritz et al., 2016). It is thus reasonable to assume that, in the context of this exercise-based music experiment, participants perceived the musical feedback compositions as rather enjoyable and motivational. Note that previously conducted experiments with a similar design (Fritz et al., 2013a,b, 2015, 2016) used similar parameters with respect to tempo (130 bpm), beat (4:4), and instrumentation (which included drums, bass line). While these parameters could certainly be varied, we decided to keep them consistent with those used in previous studies.

### Cold pressor test (CPT)

Pain tolerance was measured using the cold pressor test (Turk et al., 1984; Hsieh et al., 2010). In this test, participants placed their non-dominant hand and lower arm into cold water for as long as they could tolerate it. The time until withdrawal was used as a measure of pain tolerance. Water entry and withdrawal times were recorded with a stopwatch (values rounded to the nearest hundredth of a second). Accuracy was facilitated by a mechanical lever in the water that responded to the weight of a participant's hand by producing a clear visual signal. To prevent tissue damage, the CPT was always stopped after 5 min. The test apparatus itself consisted of a water tank with a built-in refrigeration unit that was adjusted to cool the water to 2°C. Water temperature was measured immediately after the procedure to account for the temperature variations that occur as a result of the cooling process (which uses a thermostat to switch water cooling on and off) as well as changes caused by the procedure itself (e.g., warming of the water caused by the temperature and surface area of the submerged limb).

### Pain sensitivity questionnaire

Individual differences in sensitivity to pain in everyday life were assessed using the Pain-Sensitivity-Questionnaire (PSQ; Ruscheweyh et al., 2009). The PSQ consists of 17 items (14 pain related and 3 non-pain related), each comprised of a statement describing a painful situation (e.g., pricking your fingertip on a thorn), followed by a rating of projected pain intensity on an eleven-point scale (ranging from 0 “no pain at all” to 10 “most severe pain imaginable”). The PSQ consists of two subscales, the PSQ-minor and the PSQ-moderate, which divide the items into those describing mildly painful (mean rating <4) and moderately painful situations (mean rating 4–6). PSQ scores have been shown to have high internal consistency and are independent of age and gender (Ruscheweyh et al., 2009).

### Procedure

Upon arrival at the lab (Max Planck for Human Cognitive and Brain Sciences) for the first session, each participant pair was given a brief description of the procedure during the study before providing written informed consent. The description included that they would perform physical workouts in two sessions, each with a duration of 10 min, and that they could abort the experiment at any time if they felt unwell. They were then allowed to test the fitness machines (without music) to decide who would use which machine. The machines were placed facing each other so that the participants could see each other during the exercise. For the lat-pulldown machine the participant could decide how much weight, 10 or 15 kg, felt comfortable—this was a procedure that in previous studies was successfully used to individually adapt the workout to differences in muscle strength between participants (Fritz et al., 2013a,b, 2016). Participants were required to use the same machine and the same amount of weight in both conditions. The experimenter demonstrated how to use the machines safely, and in the *agency* condition also briefly showed how the movements were mapped to the musical sounds. The participants then exercised using the machines for 10 min. Although this type of exercise is somewhat uncommon, in previous experiments it has been successfully applied to evoke positive psychological and physiological effects in participants (Fritz et al., 2013a,b, 2016). It has furthermore been argued that when associated with musical feedback it may constitute a more healthy fitness machine workout (Fritz, 2017). Immediately following completion of the exercise, participants were taken one at a time into a separate room for CPT administration. The order in which the members of each pair took the CPT was the same for both conditions. Following completion of the CPT in the first session, participants completed the PSQ. The choice to administer the PSQ at the end of the session as opposed to the beginning was made because we wanted to avoid any potential include of participant's reflecting on painful situations on their behavior in the CPT.

### Data analysis

*CPT:* The CPT data were analyzed using SPSS (version 22). A two-tailed independent samples *t*-test with the water temperature values after the *agency* and *no-agency* conditions was calculated to examine the possibility of significant differences between conditions. An analysis of covariance (ANCOVA) was performed to compare pain sensitivity between the *agency* and *no-agency* conditions (repeated measures). The PSQ-minor scores were included as a covariate in this analysis because of their previously established relationship to experimental pain ratings, and because individual differences in pain sensitivity are likely to modulate the hypoalgesic effects of with exercise through differences in the activity and effects of endogenous opioid systems (see Introduction). Gender and the order of conditions were also included in the model as between-subjects factors. Data were not included in the statistical analysis if participants did not participate in the second experimental session (on the second day), the cooling-system for the cold pressor task failed, or they completed the cold pressor test without reporting pain (to prevent tissue damage the CPT was stopped after 5 min).

### Movement data

Movement related weight shift data was extracted from the MIDI signals produced by the physical exercise machines. For each 10 min exercise session, an array of weight shift data comprising values between 0.00 and 1.00 (corresponding to the MIDI value range of 1–127), and timestamps between 0 s and 600 s (10 min) was recorded with a temporal resolution of 265.6 samples per second.

The total distance of how much the respective weight on each machine was shifted was calculated with the formula:

$$\begin{array}{l} \sum_{n=1}^{v_{max}}|v_{n-1}-v_{n}| \end{array}$$

where the parameter *v* corresponds to weight position (0.00–1.00). Weight shift distance here is an abstract value and without unit. Weight shift distance was compared between the *agency* and *no-agency* conditions for each participant.

## Results

Three of the 22 participants were excluded from the analysis (see Methods/Participants). Accordingly, the statistical analysis included 19 participants (10 male; mean age = 25.11 years; age range = 21–29; *SD* = 2.45).

### Pain tolerance

**Figure 2:**
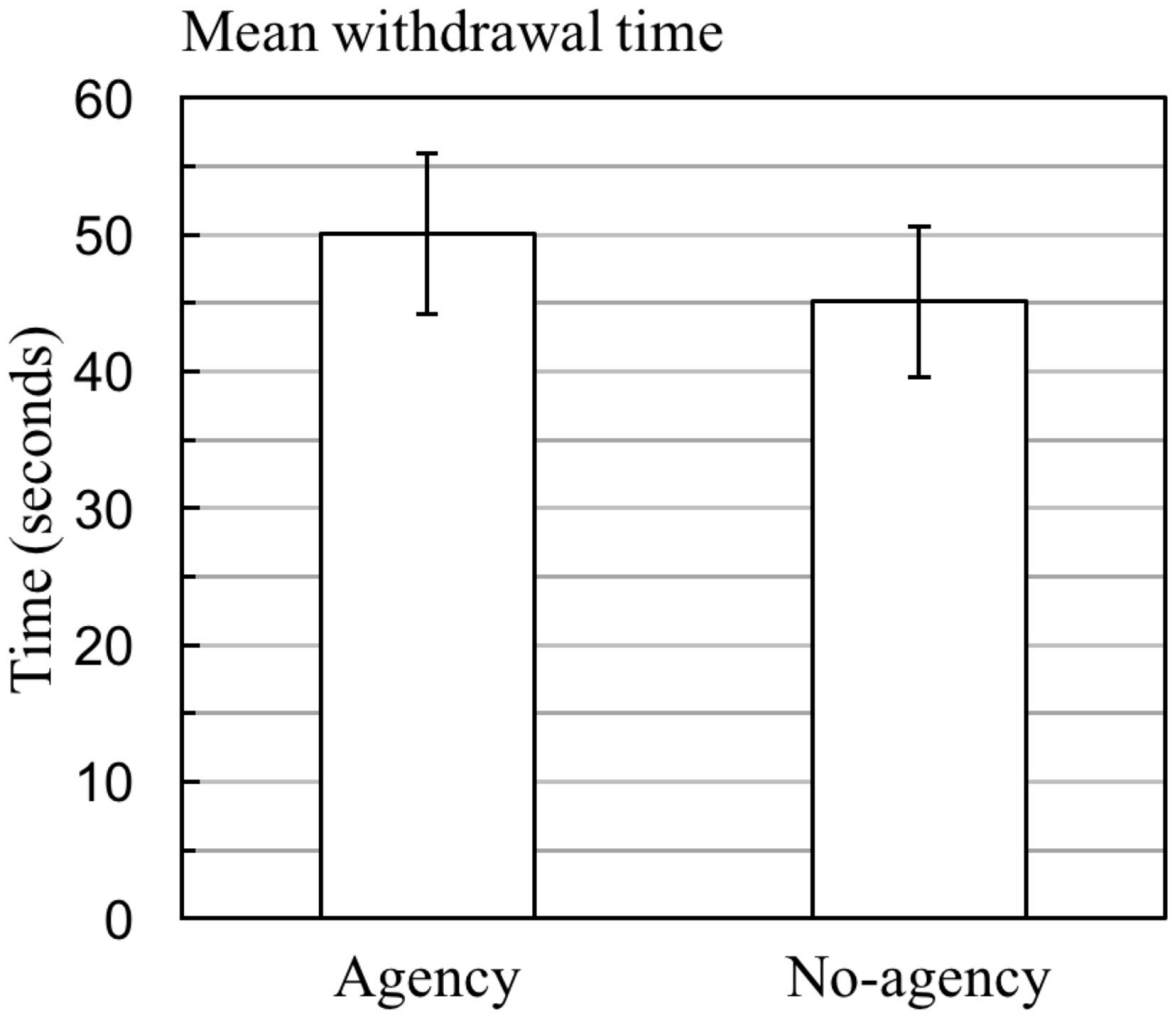
Pain tolerance following exercise with and without musical agency. Mean withdrawal times following exercise with and without musical agency (error bars represent ± SE). Statistical significance was assessed by the ANCOVA described in the main text. In the final sample, 13 out of the 19 participants started with the musical *agency* condition.

The results of the pain tolerance assessments following the *agency* and *no- agency* conditions are shown in Figure 2. On average, participants tolerated cold stimulation for 45 s following the *no-agency* condition (*SD* = 24) and 50 s following the musical *agency* condition (*SD* = 26 s). Comparison of individual pain tolerance scores with the results of the PSQ showed a significant correlation with the PSQ-minor subscale scores (*r* = −0.505, *p* = 0.027). The difference in pain tolerance between the *agency* and *no-agency* conditions was thus assessed for statistical significance using a repeated measures ANCOVA, in which PSQ-minor scores were included as a covariate. Gender and the order of conditions were also included in the model as between-subjects factors. The results showed significant effects of musical agency [*F*_(1, 13)_ = 5.974, *p* = 0.028, η^2^ = 0.250] and PSQ-minor score [*F*_(1, 13)_ = 4.652, *p* = 0.049, η^2^ = 0.236] on pain tolerance. This indicates that 23.6% of the total variance in pain tolerance was explained by participants' baseline pain sensitivity (as assessed by the PSQ-minor), and 25.0% of the residual variance was explained by the experimental manipulation of musical agency, with higher pain tolerance following the agency condition. Neither gender nor the order of conditions had significant effects on pain tolerance [*F*_(1, 13)_ = 0.087, *p* = 0.772; and *F*_(1, 13)_ = 0.029; *p* = 0.867 respectively].

### Movement data

Movement related weight shift data were analyzed separately for the two different exercise machines, the abdominal trainer and lat-pulldown machine. Average weight shift distance for the abdominal trainer was slightly higher in the *no-agency* condition than in the *agency* condition (*N* = 10, mean = 165.07, *SD* = 60.01; and *N* = 11, mean = 118.24, *SD* = 56.04 respectively), but this difference was not significant [*t*_(19)_ = −1.849, *p* = 0.080 (two-tailed), 95% CI = (−99.83, 6.17)]. For the lat-pulldown machine, however, average weight shift distance was determined to be significantly greater in the *no-agency* compared to *agency* condition [*N* = 11, mean = 85.88, *SD* = 57.09; and *N* = 10, mean = 194.81, *SD* = 74.55 respectively; *t*_(19)_ = −3.781, *p* = 0.001 (two-tailed), 95% CI = (−169.23, −48.62)].

### Water temperature

The two-tailed independent samples *t*-test with temperature values after the *agency* and *no-agency* conditions showed a significant difference [*t*_(32)_ = −3.049, *p* < 0.005, 95% CI = (−1.28, −0.25)]. Closer inspection showed that the mean water temperature in the CPTs was lower after the *agency* (*mean* = 1.56°C, *SD* = 0.81) than the *no-agency* condition (*mean* = 2.33°C, *SD* = 0.65).

## Discussion

The results of this experiment demonstrate that systematically coupling physical exercise to music making enhances pain tolerance compared to physical exercise while simply listening to music. While passive music listening has previously been shown to have the capacity to already increase pain tolerance, we here show a greater effect of active musical engagement that is specific to having agency over sound. The CPT withdrawal times and the PSQ-minor results corroborated each other in that participants who tolerated the cold water for longer durations also tended to rate questionnaire items as less painful. Factoring the PSQ-minor results into the analysis of CPT withdrawal times allowed us to separate variance attributable to individual differences in generalized baseline pain sensitivity from variance attributable to the experimental manipulation of musical agency. Results suggest that in participants with high baseline pain tolerance, the analgesic effects of the intervention have a greater positive impact, and could therefore in such individuals more strongly ameliorate quality of life and to avoiding the development of chronic pain. Given that individuals who cite pain as a barrier to do physical workout will likely have low baseline pain tolerance, it remains to be examined in training studies with a longer horizon if they can derive a benefit from the intervention.

Amount of physical activity (workload) has previously been shown to be positively correlated with CPT withdrawal times, probably due to a greater release of endorphins in conditions with greater physical exertion (Sforzo, 1989; Droste et al., 1991; Goldfarb and Jamurtas, 1997; Boecker et al., 2008). In the current experiment, workload (assessed terms of weight shift distance) was determined to be significantly greater in the *no-agency* condition, although only significantly so for the lat-pulldown machine. Critically, this indicates that the longer CPT withdrawal times associated with the musical agency condition are unlikely to be explained as the result of higher exertion levels stimulating greater opioid release. On the contrary, the longer CPT times following the *agency* condition appear to have occurred despite lower levels of exertion. This provides strong evidence that the combination of physical exercise with musical feedback is highly effective in stimulating hypoalgesia, and does so beyond physical exercise with passive music listening. The current data furthermore support previous findings suggesting that biceps training can have hypoalgesic effects (Naugle et al., 2012), and for the first time provide evidence that abdominal training can have a hypoalgesic effect.

Movement measurements showed that for the lat-pulldown machine average weight shift distance was greater in the *no-agency* compared to *agency* condition. This corresponds to the more stereotypical movement pattern exhibited by the performers during the no-agency condition, which has been shown to comprise a greater degree of isotonic movement and a smaller degree of isometric movement than in the agency condition (Fritz et al., 2013a).

Temperature measurements showed that the water was significantly colder after the CPT in the *agency* compared to *no-agency* condition (by −0.77°C). This could be due to temperature variations resulting from the nature of the cooling process (which entailed a thermostat switching water cooling on and off), or because the limbs of participants in the *no-agency* condition were at a higher temperature than those in the *agency* condition as a result of higher exertion levels. Regardless of the cause of this difference, it has been shown that even small differences in water temperature can have large effects on pain intensity and pain tolerance such that lower temperatures result in higher pain intensity and lower tolerance (Mitchell et al., 2004). Critically, the temperature data provide no support for the possibility that the longer CPT withdrawal times associated with the *agency* condition here can be explained by colder water. In fact, it is more likely that the opposite is true. Despite the water temperature being lower after the *agency* condition, which one would expect to result in greater pain intensity and lower tolerance, the opposite was the case: participants after *agency* actually remained in the water for longer.

Given the medical link between opioids and analgesia (Zubieta et al., 2001, 2003; Hsu et al., 2013), as well as the invasive and ethically more problematic nature of procedures for measuring endogenous opioid activity directly (e.g., PET scanning or lumbar puncture; Weinstein et al., 2016), measures of pain sensitivity have become a common proxy in human behavioral research (Cohen et al., 2010; Dunbar et al., 2011). Nevertheless, it is important to emphasize that although the enhancement of pain tolerance following exercise with musical agency suggests a role for endogenous opioids mechanisms in mediating the positive effects of musical agency, caution is warranted regarding this interpretation because opioid activity was not directly observed or manipulated. There are non-opioidergic mechanisms through which analgesia can arise (Bandura et al., 1987; Hebbes and Lambert, 2013) and the perception of pain is also mediated by a host of contextual factors, including attention, motivation, expectation, and emotional state (Fields, 2000; Rainville, 2002; Mannion et al., 2007; Wiech et al., 2008).

Beyond these remaining mechanistic questions, however, the fact that musical agency can be applied to reduce pain associated with physical exercise has immediate consequences for sports medicine as well as clinical injury prevention and rehabilitation. In many cases, physical exercise is the most effective form of treatment for patients suffering from physical injury (e.g., musculoskeletal damage, burn damage, and recovery after surgery) as well as neurological damage/disorders (e.g., stroke, spinal cord injury, fibromyalgia and chronic pain). In such cases exercise restores strength, increases range of motion and improves quality of life (Mior, 2001; Edgar and Brereton, 2004; Jacobs and Nash, 2004; Kwakkel et al., 2004; Houglum, 2010; Busch et al., 2011; Kroll, 2015). But pain can present a significant obstacle to the success of physical exercise rehabilitation, increasing negative affect and decreasing patient motivation (Jack et al., 2010). The capacity of musical agency to reduce pain, reduce sense of exertion (Fritz et al., 2013a) and improve mood (Fritz et al., 2013b) is thus likely to increase patient motivation and commitment, ultimately contributing to rehabilitation success. Secondary benefits include a decreased requirement for external encouragement and supervision and increased therapeutic efficiency. Musical feedback provides real-time encouragement, guiding the attention of patients toward targeted muscles and movements, increasing efficiency (Fritz et al., 2013a) and potentially facilitating the recovery of optimal control at the neuromuscular level. Finally, musical feedback technology is flexible and cost-effective (given that it can be used without constant supervision by a therapist). The only requirements are a computer, music production software, position sensors, and standard exercise equipment.

A limitation to the current study is that the age range investigated in the present study is not representative of the age range most relevant in the rehabilitation context. Further experiments may investigate if similar results can be found in a cohort of elder participants. Another limitation to the current study may be that strictly speaking we have investigated pain sensitivity immediately after the sports workout, and not during the sports workout. However, in a rehabilitation context where participants may experience pain due to physical training, it would be most relevant to quantify pain levels during the fitness workout. Tests during workouts are problematic, however, because attention to task performance is likely to be a confounding parameter. Another potential limitation is that we did not assess differences in how the participants in our study generally respond to music and/or use music during exercise. Note, however, that because the music was similar in both conditions, differences in individual response to music are unlikely to have been a confounding factor. Furthermore, it would be interesting to assess how much control each individual perceives to have over the music during the *agency* condition, and how this relates to the magnitude of the observed effects. While the percept of control should always be higher during the *agency* condition, this may vary between individuals, for example in relation to their musical skills or auditory processing capabilities. In addition, in regard of literature on Self Determination Theory and autonomy (Deci and Ryan, 1985; Ryan and Deci, 2000) we have come to the conclusion that our classification of agency has to be regarded as rather rough. For future studies a more elaborate assessment of the Self-Determination Continuum will be beneficial, especially with respect to how much the activity is perceived as extrinsically or intrinsically motivated (Ryan and Deci, 2000). Note that participants were tested in pairs, and thus influenced the music together with their actions. Their physical exercising has accordingly been a joint action, such that the experimental situation had a social aspect, which was not systematically investigated in the current study, but may have had an important influence on the shift in pain sensitivity as a result of the musical agency condition. It is a limitation that the current experiment has an imbalance of order in which participants undertook the conditions. While counterbalancing in the planning and scheduling of the experiment was accurate, unreliability on behalf of several participants (who had to come in on several days to do different conditions) required an adaptation of testing schedule, creating the imbalance. We here also want to emphasize the necessity to use cold pressor equipment that ensures a highly precise constant temperature of circulating water, as has previously been argued (Mitchell et al., 2004). While it can be a challenge to precisely control for water temperature throughout the measurement procedure, especially when the time of CPT intervention is not flexible but has to be performed at exact time points (in the current study at the end of each physical exercise intervention). This is of essential importance because the effects of the *agency* condition on pain would have been irrevocably confounded if the water temperature had by chance turned out to have been lower after the control condition. Finally, because the current paradigm used recordings of the *agency* condition in the *no-agency* condition, the first group listened to the music they created in the agency condition twice. This is relevant because familiarity with music can lead to a greater appreciation of the same style of music (North and Hargreaves, 1995). Note, however, that this was only the case for the first pair of participants and that these participants (#1 and 2; see Supplementary Table 1) did not show striking differences such that in this group the difference between *agency* and *no-agency* were not smaller than in the other groups).

At a broader level, the positive effects of musical agency may be deeply rooted in human biology. Almost all cultures exhibit some form of movement coupled to music, typically in the context of ritual or celebration (McNeill, 1997; Brown and Jordania, 2013). On such occasions, music and dance can persist for many hours and in some cases even days (McNeill, 1997). Paradoxically, such experiences tend to be associated with euphoria rather than exhaustion, suggesting that they engage powerful neurophysiological mechanisms capable of modulating bodily perceptions, leading to greater physical endurance. Using Jymmin technology and musical agency in high performance sports has the potential to increase individual endurance, using it in clinical rehabilitation therapy has the potential to harness these mechanisms for patient health and well-being.

## Author contributions

TF, LS, JG, and AV: Study design; AL and FH: Data acquisition; OC, LS, and EB: Analysis; TF and DB: Manuscript writing.

### Conflict of interest statement

The Max Planck Society has applied for intellectual property for specific aspects of the Jymmin technology at the European and U.S. patent offices, and has registered Jymmin as a trademark.
