# Supplementary material for: Musical Agency during Physical Exercise Decreases Pain
Source: Front Psychol. 2018 Jan 17;8:2312. doi: 10.3389/fpsyg.2017.02312 (PMC5776142; doi:10.3389/fpsyg.2017.02312)
Supplement: Supplementary file 1 [file Table1.DOC]

**RUNNING TITLE: Hypoalgesic effects of music**

**TITLE: Musical agency during physical exercise decreases pain**

**AUTHORS:** Fritz, Thomas Hans* ^1,2,3^, Bowling, Daniel Liu^4^, Contier, Oliver^1^, Grant, Joshua^1^, Schneider, Lydia^1^, Lederer, Annette^1^, Höer, Felicia^1^, Busch, Eric^1^, Villringer, Arno^1^.

**AFFILIATIONS:**

^1^Max Planck Institute for Human Cognitive and Brain Sciences, Stephanstrasse 1A, 04103 Leipzig, Germany, ^2^Department of Nuclear Medicine University of Leipzig, Liebigstrasse 18,  04103 Leipzig, Germany,^3^Institute for Psychoacoustics and Electronic Music (IPEM), Blandijnberg 2, B-9000 Ghent, Belgium, ^4^Department of Cognitive Biology, University of Vienna, Althanstrasse 14, 1090 Vienna

*Corresponding author: [fritz@cbs.mpg.de](mailto:fritz@cbs.mpg.de), Stephanstrasse 1A, 04103, Leipzig, Germany

| *Supplementary Table 1.: PSQ-minor scores and pain tolerance [s] of participants in the two experimental conditions* | | | | |
| --- | --- | --- | --- | --- |
|  | |  | *Pain tolerance* | |
|  | | *PSQ-minor* | *Musical agency* | *Passive listening* |
| Participant number | 1 | 3,57 | 42,24 | 37,45 |
|  | 2 | 3,14 | 56,46 | 40,44 |
|  | 3 | 2,14 | 102,33 | 95,40 |
|  | 4 | 4,00 | 37,78 | 43,19 |
|  | 5 | 2,29 | 72,32 | 92,65 |
|  | 6 | 1,43 | 55,16 | 34,70 |
|  | 7 | 1,71 | 39,13 | 17,80 |
|  | 8 | 1,57 | 37,50 | 36,13 |
|  | 9 | 4,14 | 24,85 | 31,10 |
|  | 10 | 2,14 | 71,79 | 65,96 |
|  | 11 | 3,00 | 55,51 | 43,40 |
|  | 12 | 2,29 | 39,11 | 27,74 |
|  | 13 | 2,86 | 19,56 | 22,56 |
|  | 14 | 3,00 | 31,02 | 24,24 |
|  | 15 | 2,71 | 41,63 | 44,51 |
|  | 16 | 4,71 | 33,56 | 37,04 |
|  | 17 | 1,57 | 106,53 | 79,76 |
|  | 18 | 1,14 | 72,55 | 68,49 |
|  | 19 | 4,14 | 12,05 | 14,36 |
